# Soluble FcɛRI: A biomarker for IgE‐mediated diseases

**DOI:** 10.1111/all.13734

**Published:** 2019-03-11

**Authors:** Sherezade Moñino‐Romero, Willem S. Lexmond, Josef Singer, Christina Bannert, Abena S. Amoah, Maria Yazdanbakhsh, Daniel A. Boakye, Erika Jensen‐Jarolim, Edda Fiebiger, Zsolt Szépfalusi

**Affiliations:** ^1^ Department of Pediatrics and Adolescent Medicine Medical University Vienna Vienna Austria; ^2^ Department of Pediatrics Division of Gastroenterology, Hepatology and Nutrition Boston Children's Hospital Boston Massachusetts; ^3^ Department of Medicine Harvard Medical School Boston Massachusetts; ^4^ Institute of Pathophysiology and Allergy Research Center of Pathophysiology, Infectiology and Immunology Medical University of Vienna Vienna Austria; ^5^ The Interuniversity Messerli Research Institute of the University of Veterinary Medicine Vienna Medical University Vienna and University Vienna Vienna Austria; ^6^ Department of Internal Medicine II University Hospital Krems Karl Landsteiner University of Health Sciences Krems an der Donau Austria; ^7^ Department of Parasitology Leiden University Medical Center Leiden The Netherlands; ^8^ Department of Parasitology Noguchi Memorial Institute for Medical Research College of Health Sciences University of Ghana Legon‐Accra Ghana

**Abbreviations**
cIgE: chimeric humanized anti‐NIP immunoglobulin E
DC: dendritic cell
FcεRI: Fc epsilon Receptor I, high‐affinity IgE Fc receptor
IgE: Immunoglobulin E
IQR: interquartile range
MC: mast cell
OFC: oral food challenge
rsFcεRI^m^: mutant recombinant human sFcεRI
rsFcεRI: recombinant human sFcεRI
sCD23: soluble isoform of CD23, low‐affinity IgE Fc receptor
sFcεRI: soluble isoform of FcεRI
sIgE: allergen‐specific immunoglobulin E
SPT: skin prick test
εBP: epsilon binding protein

## CONFLICTS OF INTEREST

The authors declare that they have no conflicts of interest.

To the Editor,

Soluble IgE receptors interact with IgE in the extracellular matrix and are important in the regulation of immune diseases.^1^, ^2^, ^3^, ^4^, ^5^ Soluble FcεRII (sCD23) and galectin‐3 (εBP) are currently used as biomarkers,^1^ though correlation data on serum titers and severity of allergies are controversial.^1^, ^6^

FcεRI, the high‐affinity IgE Fc receptor, is expressed on several innate cell types,^2^ and a truncated version of the IgE‐binding alpha subunit is found as a soluble isoform (sFcεRI) in human serum. In circulation, sFcεRI is mostly detected as a complex with IgE.^7^ This observation raises the question of how sFcεRI affects detection of serum IgE titers.

In order to assign clinical implications of sFcεRI, we assessed serum titers in its total and IgE‐bound forms in different IgE‐mediated diseases in 312 individuals. We compared pediatric populations with primary food allergies (n = 59), insect venom allergies (n = 9), allergic asthma (n = 24), atopic dermatitis (n = 25), food‐sensitized nonallergic children (n = 31), and nonallergic controls (n = 17). Additionally, other sensitized groups and controls (n = 147) were included in the study (Table S1‐S4).

## sFcεRI IS ELEVATED IN SERUM OF ATOPIC INDIVIDUALS AND IS MODULATED BY ALLERGEN EXPOSURE

**Figure 1:**
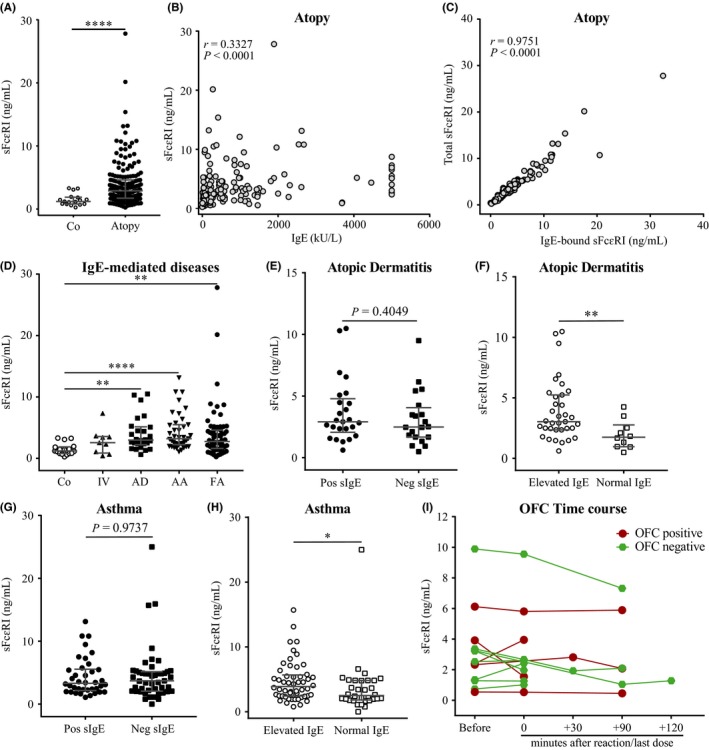
sFcεRI is highly expressed in allergic individuals and it is modulated by allergen exposure. Detection of total and IgE‐bound sFcεRI levels by ELISA. Total sFcεRI levels in control and atopic (n = 148) groups (A). Correlation between total sFcεRI and total IgE levels in atopic group (B). Total and IgE‐bound sFcεRI levels in atopic group (C). Total sFcεRI levels in control and IgE diseases groups (D). Total sFcεRI levels with and without sIgE sensitizations, and normal and elevated IgE levels in AD (E‐F) and AA (G‐H). Total sFcεRI levels during OFC (I). Graphs represent individuals with median plus IQR. Mann‐Whitney test (A, E‐H), Kruskal‐Wallis test plus Dunn's multiple correction (C), and Spearman r coefficient ranks (B, D) were performed, where *P < 0.05, **P < 0.01, and ****P < 0.0001. Co: control (n = 17); IV: insect venom (n = 9); AD: atopic dermatitis (n = 45); AA: allergic asthma (n = 69); FA: food allergy (n = 59); Pos: positive; Neg: negative; IQR: interquartile range; OFC: oral food challenge (n = 13) [Colour figure can be viewed at wileyonlinelibrary.com]

Serum samples were analyzed by ELISA to detect IgE‐bound and total serum sFcεRI levels (Figure S1). First, sFcεRI was ubiquitously detectable among controls (median 1.20 ng/mL) but titers were significantly higher in atopic individuals (median 2.88 ng/mL, Figure 1A and Table S1). In line with previous studies,^7^, ^8^ IgE and sFcεRI levels correlated positively in all patients, and sFcεRI in circulation was almost uniquely detected as a complex with IgE (Figure 1B,C). Next, we grouped the atopic individuals based on their main IgE‐mediated disease (Table S2) as food allergy (FA), insect venom allergy (IV), allergic asthma (AA), or atopic dermatitis (AD). AD, AA, and FA groups presented with significantly higher sFcεRI levels than controls (Figure 1D).

Since IgE‐sensitization profiles toward food allergens are generally a poor measure of clinical symptoms, we compared sFcεRI titers in two food‐sensitized nonallergic groups (FS and Ghana) with FA patients (Table S3). The Ghana cohort showed similar correlations as already described between IgE and sFcεRI, IgE‐bound and total sFcεRI levels, and no correlation with peanut‐specific IgE (sIgE) titers. No significant difference was detected with regards to disease activity among food‐sensitized individuals (Figure S2).

We then investigated whether serum sFcεRI levels were different in patients diagnosed with atopic dermatitis or asthma, with (Pos sIgE) or without (Neg sIgE) a clinically relevant sIgE profile. sFcεRI titers did not differ based on the patients’ sIgE profile. However, we found significantly higher titers in patients with elevated IgE (Figure S3) in both AD and AA groups (Figure 1E‐H).

Recently, we demonstrated that sFcεRI is released from dendritic cells and mast cells after antigen‐specific FcεRI crosslinking.^5^ Thus, we studied how sFcεRI levels in circulation are affected by allergen exposure. We compared sFcεRI levels in AA individuals (n = 14 pairs) during (In) and before/after (Out) season for their most clinically relevant allergen (Table S4) and observed that serum levels could significantly increase (50%) or decrease (50%) during season. This pattern was similarly observed with total IgE levels (Figure S4). In order to better determine the role of allergen exposure, we analyzed food‐sensitized individuals on allergen avoidance (n = 13) during an oral food challenge (Figure S5). We observed a general trend of sFcεRI titers to decrease after allergen exposure (Figure 1I).

## IgE:sFcεRI COMPLEXES INTERFERE WITH IgE DETECTION

**Figure 2:**
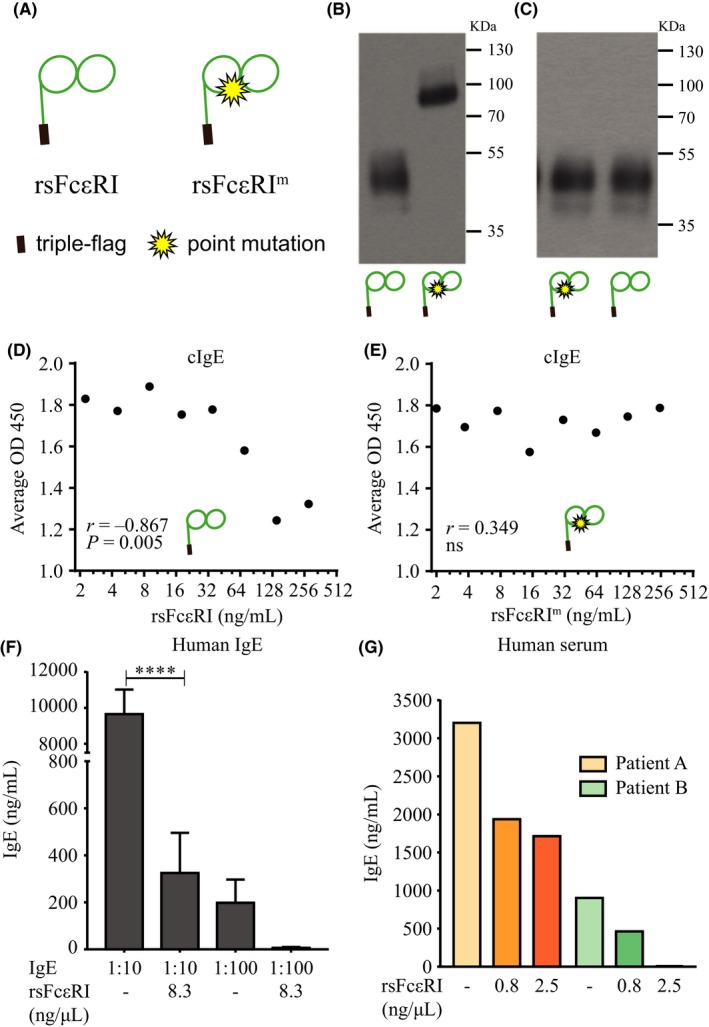
sFcεRI interferes with IgE detection ELISA. Detection of IgE and sFcεRI levels by ELISA and Western Blot. Representation of rsFcεRI and rsFcεRI ^m^ proteins (A). Detection of rsFcεRI and rsFcεRI ^m^ proteins by Western Blot analysis in nonreducing and reducing conditions (B‐C). Detection of IgE pre‐incubated with rsFcεRI and rsFcεRI ^m^ proteins in a 500 ng/mL cIgE solution (D‐E). Detection of IgE pre‐incubated with rsFcεRI in human IgE (1:10‐1:100) or human serum (3202 and 903 ng/mL) solutions (F‐G). Graphs represent assay triplicates of a representative experiment (D‐E), or assay duplicates of biological triplicates (F) or two individuals (G). Spearman coefficient rank analysis or 1‐way ANOVA test plus Tukey's multiple correction was performed, where *P < 0.05 and ****P < 0.0001

sFcεRI binds to the Fc portion of IgE and can potentially interfere with antibody binding to that region. We thus investigated whether sFcεRI affects antibody‐based IgE detection. For this purpose, a recombinant IgE‐binding protein (rsFcεRI) and a mutated version which cannot bind IgE (rsFcεRI^m^) were generated. Prior to a commercial IgE ELISA, samples containing human cIgE were incubated with the recombinant proteins (Figure 2A‐C). Our hypothesis was that IgE detection will be impaired and reflected in a decrease of IgE levels with increasing concentrations of rsFcεRI. In Figure 2D, we show an *r* = −0.867 with *P* = 0.005 which depicts a significant negative correlation in support of our hypothesis. On the contrary, as shown in Figure 2E, increasing concentrations of the mutant version of rsFcεRI which is unable to bind IgE do not show interference in IgE detection (*r* = 0.349, ns). This interference with IgE detection by rsFcεRI was confirmed with human IgE (Figure 2F) and human serum (n = 2) from patients with elevated IgE levels (Figure 2G). In addition, we observed that sFcεRI titers were significantly higher in serum than plasma (Figure S6).

To the best of our knowledge, this is the first analysis of sFcεRI levels in a pediatric population of well‐classified sensitized and allergic individuals. We show that sFcεRI is correlated with IgE levels, is significantly increased in IgE‐sensitized individuals, and can be modulated by allergen exposure. We collected evidence that sFcεRI can interfere with IgE detection in serum, which might be of importance in regard to interference in sIgE detection and diagnosis. Although further research on the modulation by allergen exposure and interference with sIgE molecules is needed, sFcεRI represents an additional biomarker for IgE‐mediated diseases and its use could be a valuable tool in clinical practice.
