# Supplementary material for: Soluble FcɛRI: A biomarker for IgE‐mediated diseases
Source: Allergy. 2019 Mar 11;74(7):1381–4. doi: 10.1111/all.13734 (PMC6766993; doi:10.1111/all.13734)
Supplement: Supplementary file 1 [file ALL-74-1381-s002.docx]

**SUPPORTING INFORMATION**

**Soluble FcɛRI: a biomarker for IgE-mediated diseases**

Sherezade Moñino-Romero^1,2^, Willem S. Lexmond^2,3^, Josef Singer^2,4,5,6^, Christina Bannert^1^, Abena S. Amoah^7^, Maria Yazdanbakhsh^7^, Daniel A. Boakye^8^, Erika Jensen-Jarolim^4,5^, Edda Fiebiger^2,3^, and Zsolt Szépfalusi^1*^

* Corresponding author

^1^Department of Pediatrics and Adolescent Medicine, Medical University Vienna, Vienna, Austria

^2^Department of Pediatrics, Division of Gastroenterology, Hepatology and Nutrition, Boston Children's Hospital, Boston, Massachusetts, USA

^3^Department of Medicine, Harvard Medical School, Boston, Massachusetts, USA

^4^Institute of Pathophysiology and Allergy Research, Center of Pathophysiology, Infectiology and Immunology, Medical University of Vienna, Vienna, Austria

^5^The interuniversity Messerli Research Institute of the University of Veterinary Medicine Vienna, Medical University Vienna and University Vienna, Austria

^6^Department of Internal Medicine II, University Hospital Krems, Karl Landsteiner University of Health Sciences, Krems an der Donau, Austria

^7^Department of Parasitology, Leiden University Medical Center, Leiden, Netherlands

^8^Department of Parasitology, Noguchi Memorial Institute for Medical Research, College of Health Sciences, University of Ghana, Legon-Accra, Ghana.

**To whom correspondence should be addressed:**

Zsolt Szépfalusi, MD, Department of Pediatrics and Adolescent Medicine, Medical University of Vienna, Waehringer Guertel 18-20, 1090 Vienna, Austria, Tel: +43 1 40400 12320; Email: zsolt.szepfalusi@meduniwien.ac.at

**ABBREVIATIONS:**

cIgE (chimeric humanized anti-NIP immunoglobulin E)

DC (dendritic cell)

εBP (epsilon binding protein)

FcεRI (Fc epsilon Receptor I, high affinity IgE Fc receptor)

IgE (Immunoglobulin E)

IQR (interquartile range)

MC (mast cell)

OFC (oral food challenge)

sCD23 (soluble isoform of CD23, low affinity IgE Fc receptor)

sFcεRI (soluble isoform of FcεRI)

sIgE (allergen-specific immunoglobulin E)

rsFcεRI (recombinant human sFcεRI)

rsFcεRI^m^ (mutant recombinant human sFcεRI)

SPT (skin prick test)

**MATERIAL AND METHODS**

**ELISAs**

Levels of total and IgE-bound soluble FcεRI (sFcεRI) in serum and plasma were measured by a commercial ELISA according to manufacturer’s protocol (BMS2101, Thermo Fisher Scientific, Waltham, MA, USA) as previously described (1, 2). Values are given in ng/mL.

Total IgE and sIgE levels from patients’ serum were measured by solid phase immunoassay (ImmunoCAP®, Phadia, Uppsala, Sweden). Total IgE values are given in kU/L, and sIgE values in kU_A_/L. Expected cut-off values for total IgE are <10 kU/L for age <1 years, <15 kU/L for age 1-7y, <25 kU/L at age 8y, <50 kU/L at age 9y, <75 kU/L at age 10-12y and <100 kU/L at age 12-16y (2). IgE levels above the age specific normal range are referred to as ‘elevated’ IgE from here on. Total IgE values below 2 kU/L were considered as 0, and values above 5000 kU/L were considered as 5000.

IgE-detection ELISA used to assess interference by sFcεRI was performed with a commercial kit for human IgE samples (BMS2097, Invitrogen, Thermo Fisher Scientific). Wild type or mutant rsFcεRI was incubated for 60 minutes in agitation at 37˚C with human serum (n= 2), human IgE (401152, Calbiochem, Darmstadt, Germany), or cIgE (clone JW8/1 Serotec BioRad, Hercules, CA, USA). Data with cIgE are assay triplicates from a selected experiment. Data with human IgE and serum are assay triplicates from biological replicates or independent experiments (n= 2-3).

**Oral Food Challenge (OFC)**

OFC were performed according to standard procedures as defined in DRACMA as previously described (25).

**Study design, patient information and classification**

Non-atopic controls (age 2-20 years old, n= 17), were defined as <0.35 kU_A_/L allergen-specific IgE (sIgE) levels, low total IgE levels, and did not refer allergic symptoms. Atopic individuals (age <20 years old, n= 150) were defined by >0.35 kU_A_/L sIgE to at least one allergen, and low or elevated total IgE levels.

Atopic non-allergic food-sensitized patients (FS, n=31) were defined by >0.35 kU_A_/L sIgE to at least one allergen and/or >3 mm wheal size in skin prick test (SPT), and no clinical symptoms or negative reaction to oral food challenge (OFC).

Allergic patients were defined by >0.35 kU_A_/L sIgE to at least one allergen and/or >3 mm wheal size in SPT, and any of the following clinical symptoms: food allergy (FA), insect venom allergy (IV), allergic asthma (AA), and atopic dermatitis (AD). Diagnosis of FA (n= 59) was defined by objective symptoms during OFC or convincing clinical history after food consumption. Diagnosis of IV (n= 9) allergy was defined by specific IgE to wasp or bee venom >0.35 kU_A_/L and clear clinical history of systemic reaction after bee/wasp sting. Diagnosis of AA (n= 24) was defined according to standardized criteria (1) and clinically relevant IgE sensitizations. Non-AA (n= 45) was defined by <0.35 kU_A_/L sIgE levels. Serum from 14 AA pairs was collected during (In) and after/before (Out) season of their most clinically relevant allergen. Diagnosis of AD (n= 25) was defined according to standardized criteria of Hanifin & Rajka (Acta Derm Venereol (Stockh) 1980; Suppl 92: 44–47) and clinically relevant IgE sensitization. AD with <0.35 kU_A_/L sIgE levels were classified as no IgE-mediated AD patients (n=20).

African populations are often studied in regards with peanut allergy due to the high prevalence of peanut sensitization and very few reports of allergic reactions after peanut consumption. For this reason, serum of individuals (n= 47) from different environments (urban high socioeconomic status, urban low socioeconomic status, and rural areas) in Ghana, West Africa, were defined by >0.35 kU_A_/L sIgE levels to at least one major peanut allergen and/or >3 mm wheal size in SPT, with no reports of clinical reaction after peanut consumption.

Serum and plasma samples collected at the same time point from atopic children (n= 35) during two independent consult visits. Serum samples were collected in serum tubes and plasma samples were collected in lithium-heparin-coated tubes.

A total of 312 individuals were used in the analysis. All patients included in the study gave written informed consent and were recruited after approved application from the Ethics Commission, Medical University of Vienna (EK Nr: 1015/2017 and EK Nr: 079/2009), and the Noguchi Memorial Institute for Medical Research Institutional Review Board, Ghana (NMIMR-IRB CPN 012/04-05).

**Expression of rsFcεRI and rsFcεRI^m^**

For expression of recombinant sFcεRI (rsFcεRI), human FcεRI-cDNA was introduced into the pCMV-3Tag-8 vector (Agilent Technologies, Santa Clara, California, USA) via the BamHI (forward) and XhoI site. This expression vector provides three FLAG^®^ epitope tags at the N-terminus. To achieve a soluble form of the normally membrane bound FcεRI-α-chain, the protein was truncated after 179 amino acids from the C-terminus. In order to investigate the specific effects of IgE binding to the sFcεRI, a loss of function-mutant was generated by introducing a Glycine instead of a Cysteine at position 128 (rsFcεRI^m^) via site-directed mutagenesis (QuikChange II Site-Directed Mutagenesis Kit, Agilent Technologies, Santa Clara, California, USA) using the primers 5'-ccctcttcctcaggggccatggttggagg-3' and 5'-cctccaaccatggcccctgaggaagaggg-3'.

HEK-293T cells were transfected using either FuGENE^®^ 6 transfection reagent (Roche Diagnostics, Indianapolis, Indiana, USA) or PEI (Polyethenylenimine, MW: 25 kDa; Polysciences Inc, Warrington, Pennsylvania, USA) according to the manufacturers’ instructions. Transfected cells were selected using hygromycin (1 mg/mL Sigma-Aldrich, St. Louis, Missouri, USA). Supernatants were harvested and the recombinant protein was purified via affinity purification using the FLAG^®^ M Purification Kit (Sigma-Aldrich).

**Cell lines and monoclonal antibodies**

Recombinant proteins rsFcεRI and rsFcεRI^m^ were produced in human embryonic kidney cells, which were obtained from the American Type Culture Collection (HEK-293T, ATCC number CRL3216™). HEK-293T cells were maintained in Dulbecco’s Modified Eagle Medium (DMEM, Cellgro, MediaTech, Herndon, Virginia, USA) supplemented with fetal calf serum (HyClone, Logan, Utah, USA) 2 mM glutamine (Cellgro), 100 U/ml penicillin, and 100 mg/mL streptomycin (Gibco, Thermo Fisher Scientific). Cells were kept under humidified conditions (37°C, 5% CO_2_).

The anti-FcεRIα antibody CRA1 was purchased from eBioscience (clone AER-37, eBioscience, San Diego, California, USA). The monoclonal antibody 19-1, also reactive against FcεRIα was kindly provided by Dr. J.P. Kinet (Laboratory of Allergy and Immunology, Beth Israel Deaconess Medical Center, Boston, Massachusetts).

**SDS-PAGE and Western Blots**

Recombinant proteins were separated on 12% SDS-PAGE gels (Precise™ Tris-HEPES Gels, Pierce Protein Biology Products, Thermo Fisher Scientific) either in reducing or non-reducing conditions, transferred to PVDF membranes (Thermo Fisher Scientific) and probed with either the anti-FcεRIα antibody CRA1, or 19-1, respectively, followed by peroxidase (HRP)-conjugated Ab detection. Additionally, membranes were incubated with the anti-FLAG monoclonal antibody M2-HRP (Sigma-Aldrich). Peroxidase activity was detected by chemiluminescent substrate (SuperSignal West Pico Chemiluminescent Substrate, Pierce Protein Biology Products).

Pre-incubation of rsFcεRI and rsFcεRI^m^ with chimeric humanized anti-NIP IgE (cIgE) (MCA333S clone JW8/1 Serotec BioRad, Hercules, CA, USA) was performed.

**Statistical analyses**

All statistical analyses were performed using Prism 7 (GraphPad Software). Some of the parameters analyzed do not show a normal distribution, for this reason all statistical analysis were performed with non-parametric tests. Correlation between total IgE and total sFcεRI levels, and IgE-bound and total sFcεRI levels were calculated by Spearman’s rank correlation test. Spearman’s rank correlation coefficients are displayed as “r”; a p-value of < 0.05 was considered significant. Wilcoxon matched-pairs test or Mann-Whitney test was performed to compare two groups, and Kruskal-Wallis test plus Dunn’s multiple correction to compare more than two groups. Assuming normality in IgE-detection interference experiments, 1way ANOVA test plus Dunn’s multiple correction analysis was performed.

**REFERENCES**

1. Monino-Romero S, Erkert L, Schmidthaler K, Diesner SC, Sallis BF, Pennington L, et al. The soluble isoform of human FcepsilonRI is an endogenous inhibitor of IgE-mediated mast cell responses. Allergy. 2018.

2. Lexmond W, der Mee J, Ruiter F, Platzer B, Stary G, Yen EH, et al. Development and validation of a standardized ELISA for the detection of soluble Fc-epsilon-RI in human serum. J Immunol Methods. 2011;373(1-2):192-9.

**
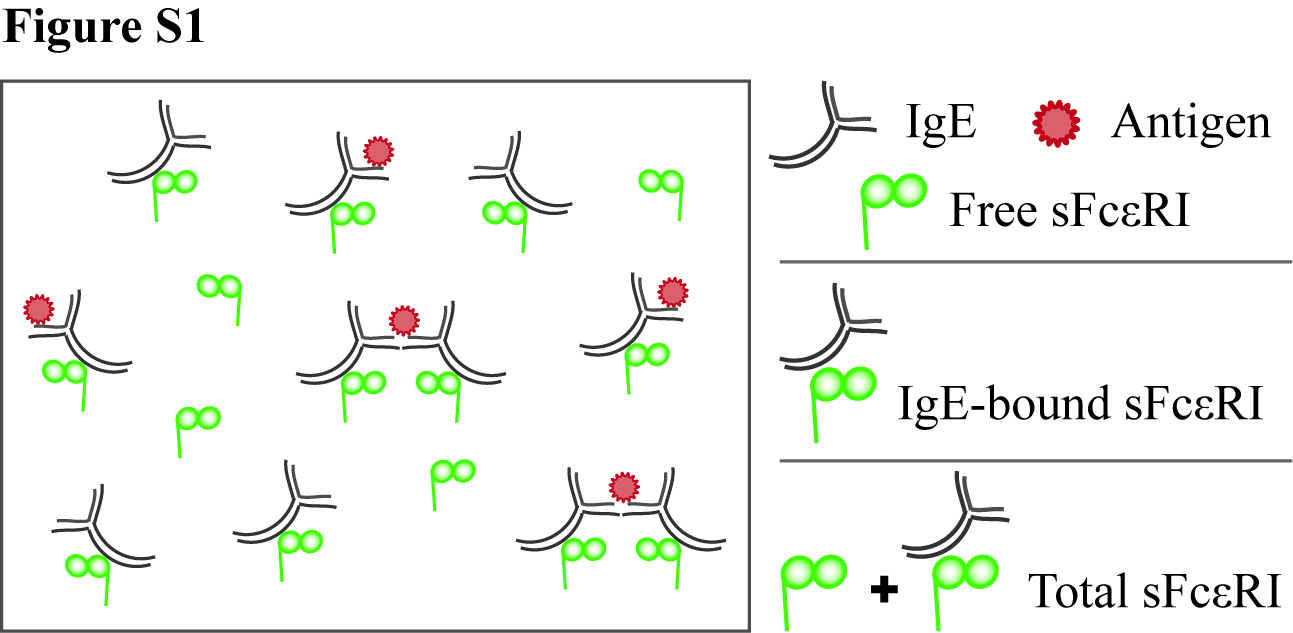
**

**Figure S1. Schematic representation of total and IgE-bound sFcεRI in circulation.** Total and IgE-bound sFcεRI detection was performed by ELISA through anti-IgE-HRP antibody.


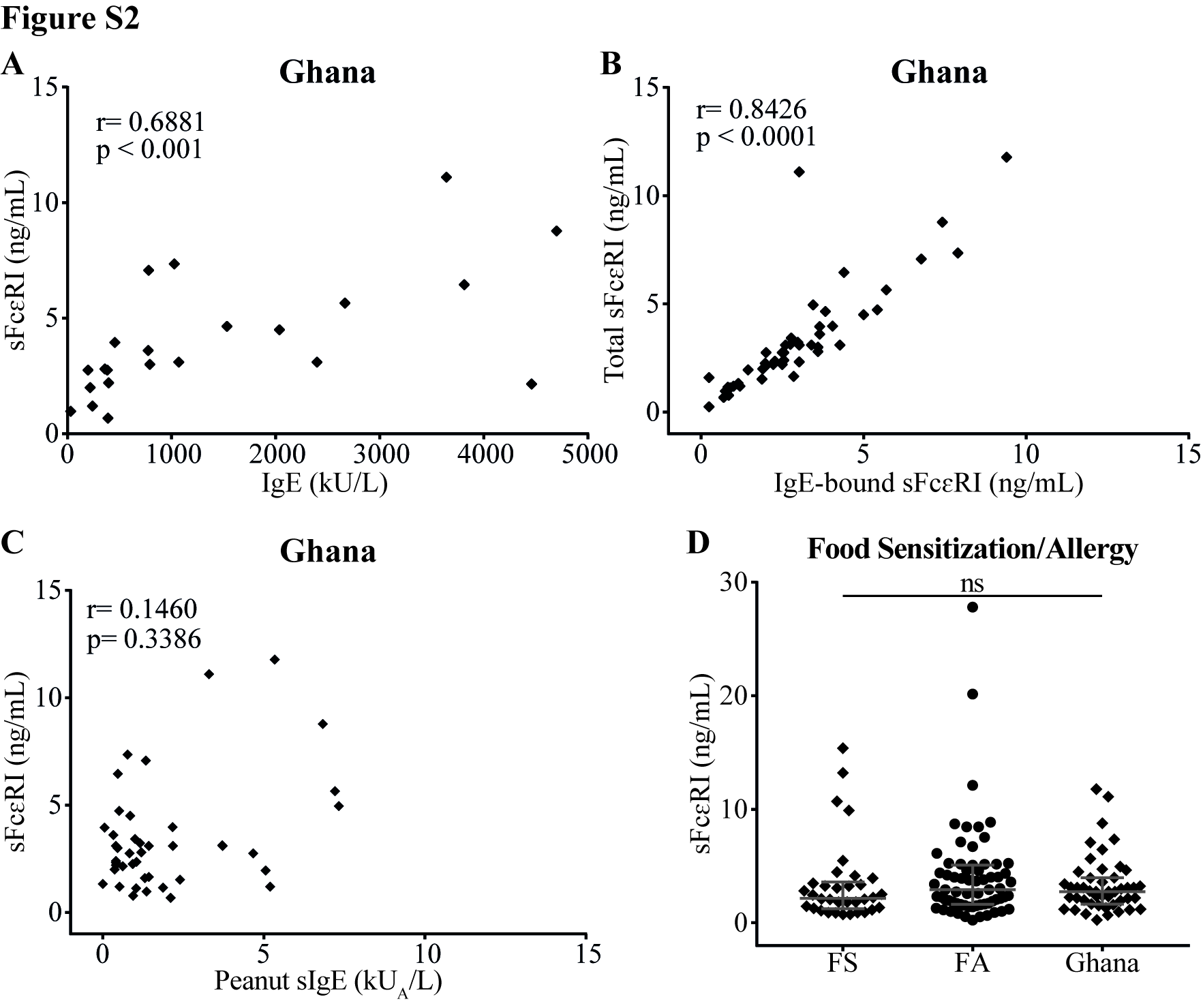


**Figure S2. Correlation between IgE and sFcεRI, and total and IgE-bound sFcεRI levels in a peanut-sensitized group from Ghana, West Africa.** Panel **A** shows correlation between total sFcεRI and total IgE levels (n= 22). Panel **B** shows total and IgE-bound sFcεRI levels (n= 47). Panel **C** shows correlation between total sFcεRI and peanut sIgE levels (n= 45). Panel **D** shows total sFcεRI levels in FS (n= 31), FA (n= 59), and Ghana (n= 47) groups. Graphs represent individuals. Spearman r coefficient rank analysis was performed. FS: food sensitization; FA: food allergy.


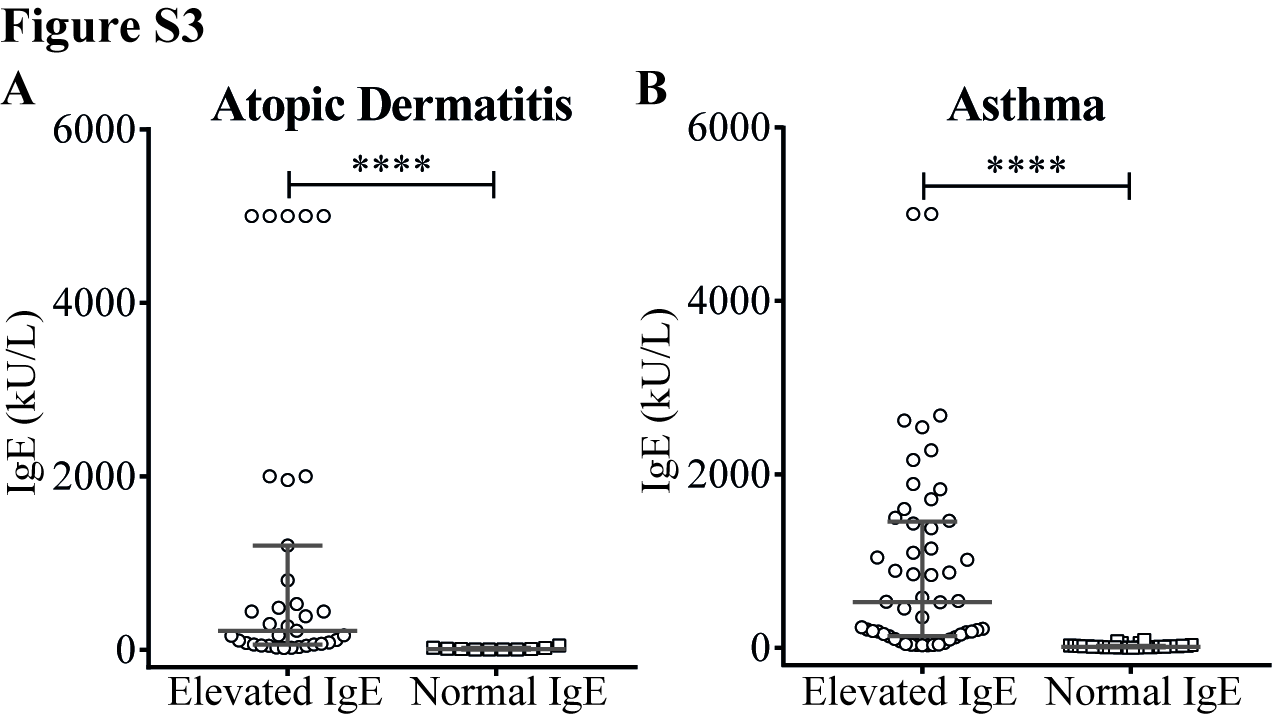


**Figure S3. Normal and elevated IgE levels in AD and AA.** Panel **A** shows total IgE levels in AD group (n= 35, n= 10). Panel **B** shows total IgE levels in AA group (n= 52, n= 32). Graphs represent individuals. Mann-Whitney test was performed, where ****p<0.0001. AD: atopic dermatitis; AA: allergic asthma.


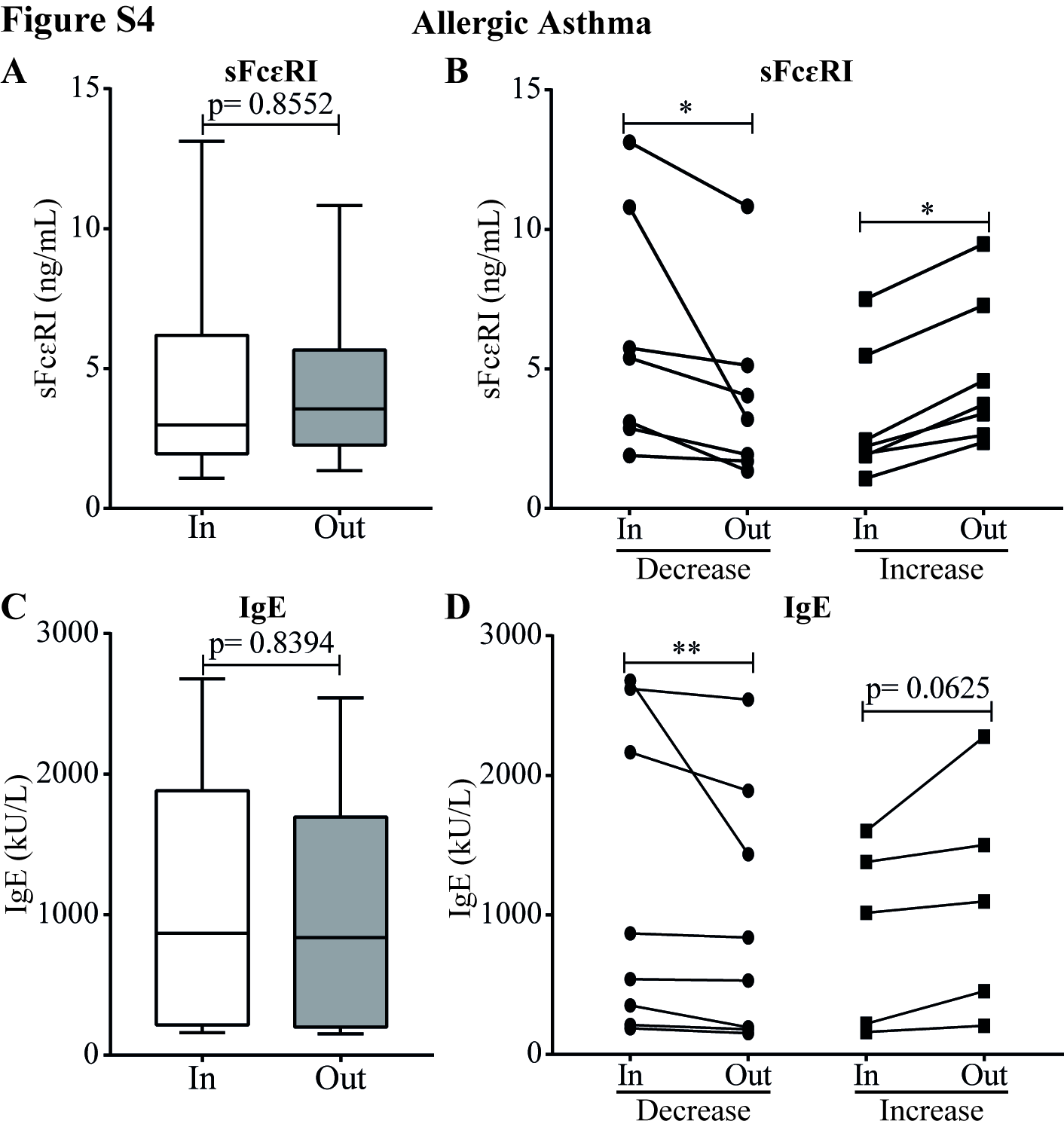


**Figure S4. sFcεRI and IgE levels are modulated by allergen exposure in AA.** Panels **A-B** show total sFcεRI levels in AA group In and Out of season combined (n= 14 pairs) and divided according to a decrease (n= 7) or increase (n= 7). Panels **C-D** show total IgE levels in AA group In and Out of season combined (n= 13 pairs) and divided according to a decrease (n= 8) or increase (n= 5). Graphs represent individuals. Paired Wilcoxon test was performed, where *p<0.05 and **p<0.01. AA: allergic asthma.


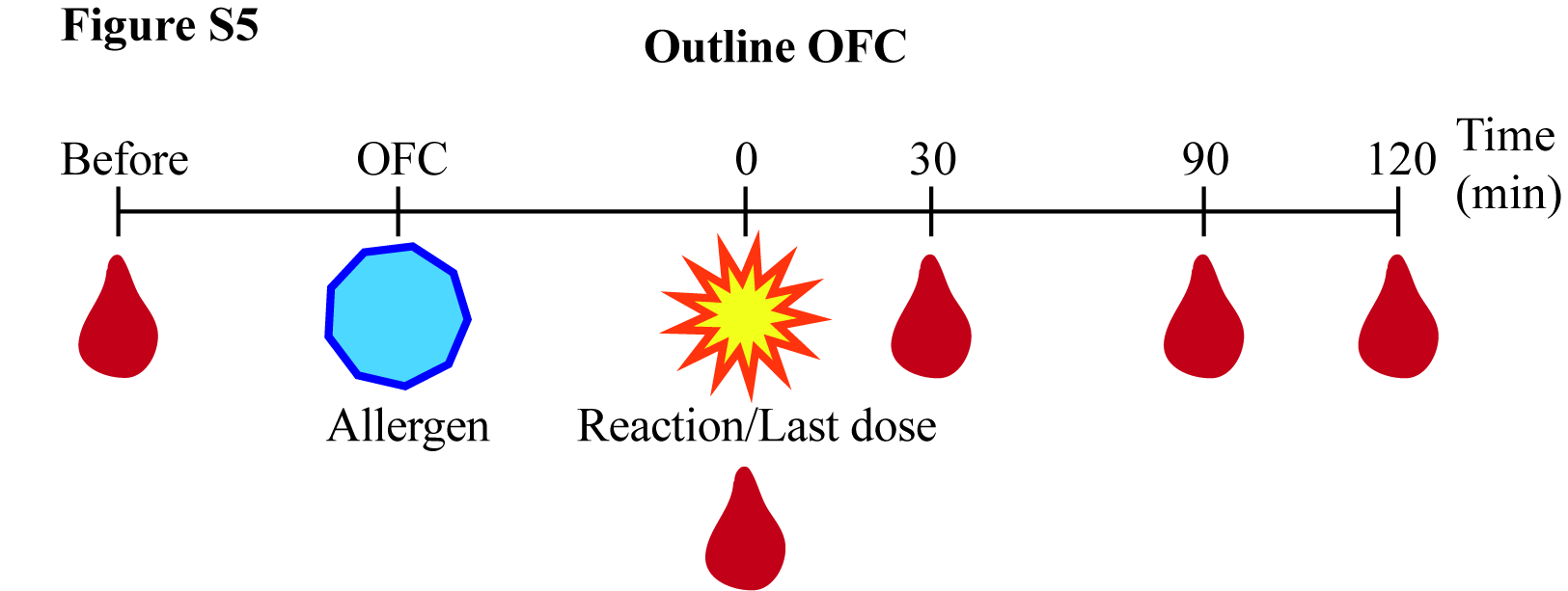


**Figure S5. Schematic outline of sampling at different time points during OFC.** Blood samples were collected from patients who undergo OFC at different time points: before, at reaction or after last dose, and after 30/90/120 minutes. Serum was isolated and harvested for sFcεRI detection by ELISA. OFC: oral food challenge.


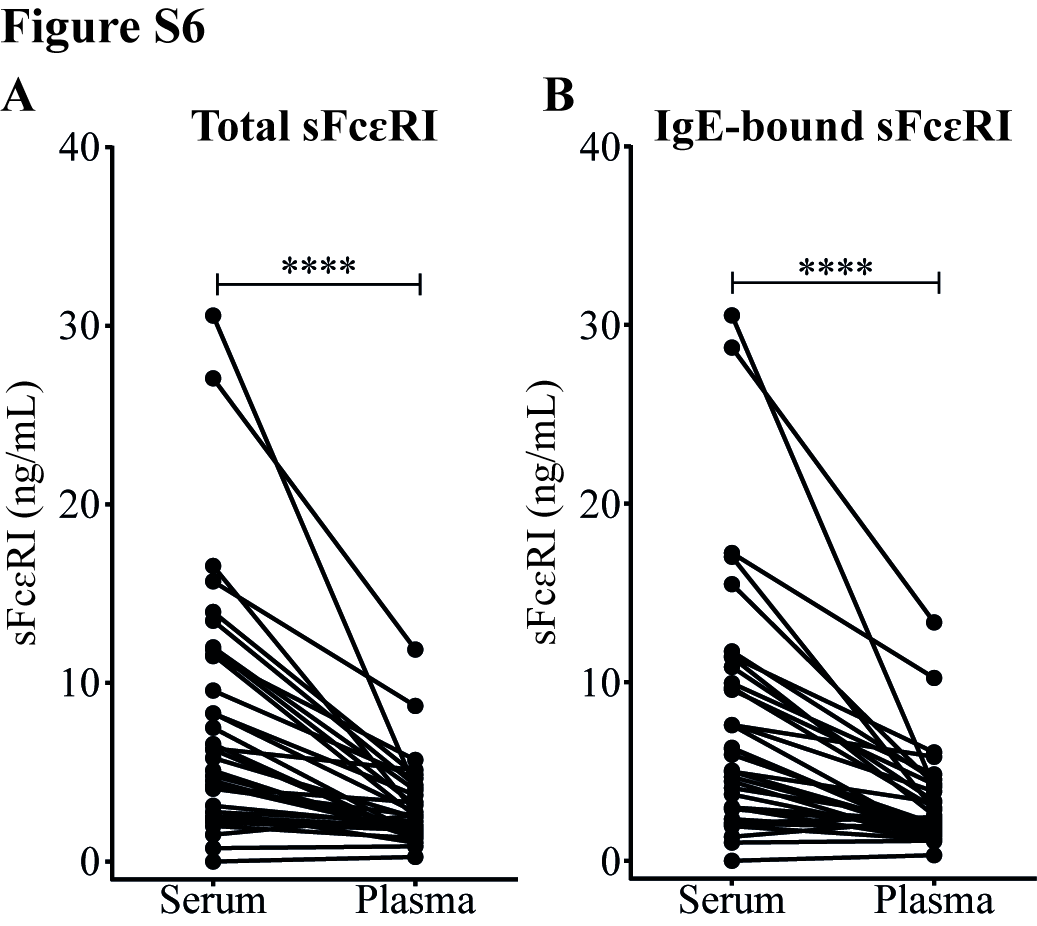


**Figure S6. sFcεRI is more stable in serum.** Detection of total and IgE-bound sFcεRI levels by ELISA. Panel **A** shows total sFcεRI levels in serum and plasma samples. Panel **B** shows IgE-bound sFcεRI levels in serum and plasma samples (n= 35). Graphs represent individuals. Wilcoxon test was performed, where ****p<0.0001.


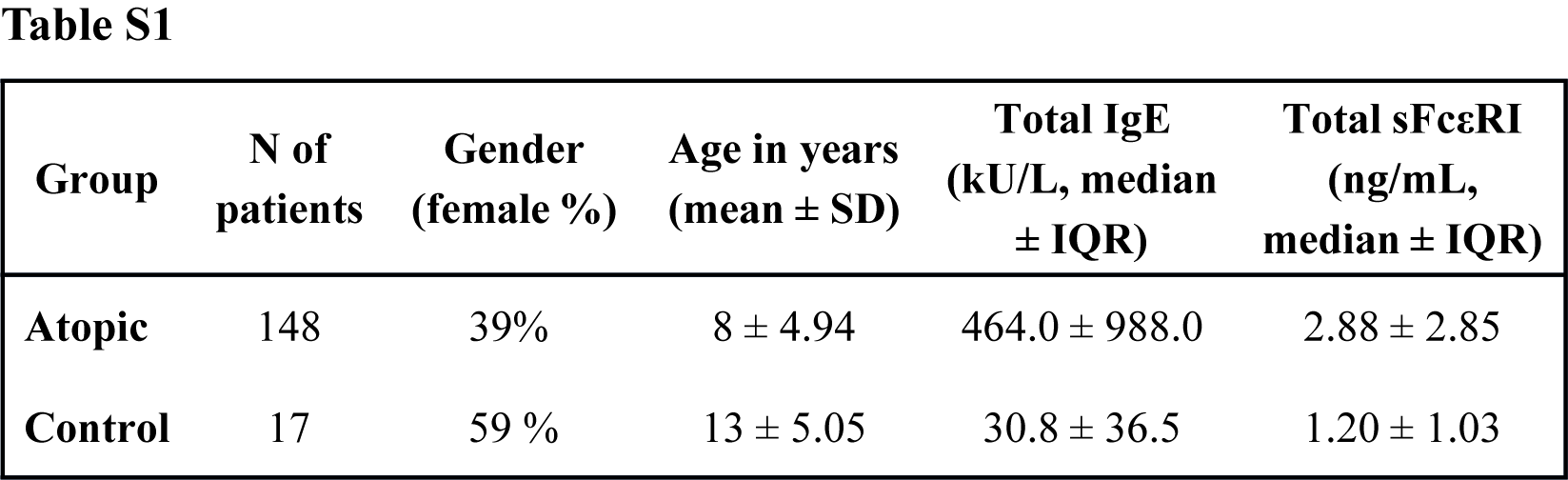


**Table S1: Patient characteristics of atopic and control groups.** SD: standard deviation; IQR: interquartile range.


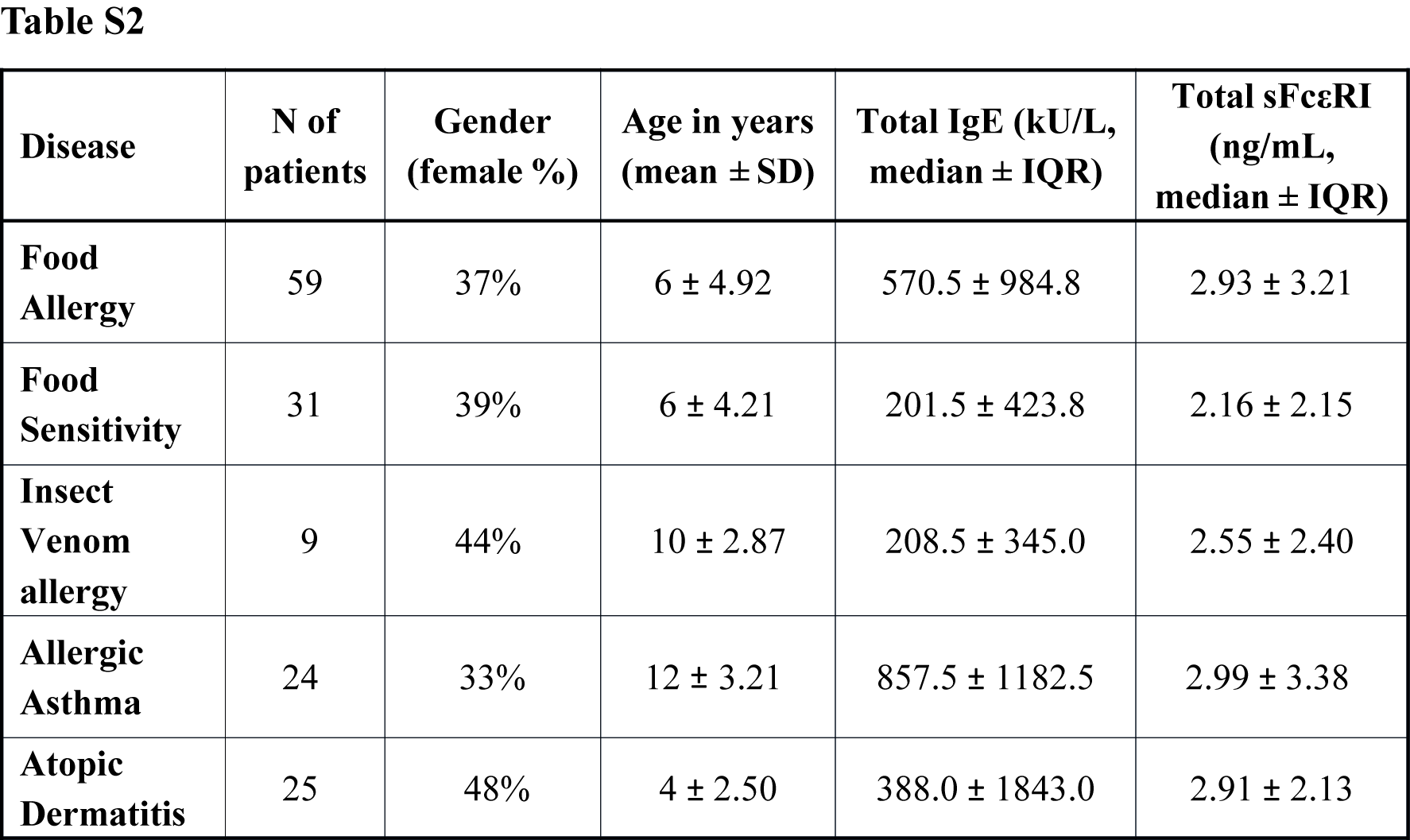


**Table S2: Patient characteristics of IgE-mediated disease groups.** SD: standard deviation; IQR: interquartile range.

**
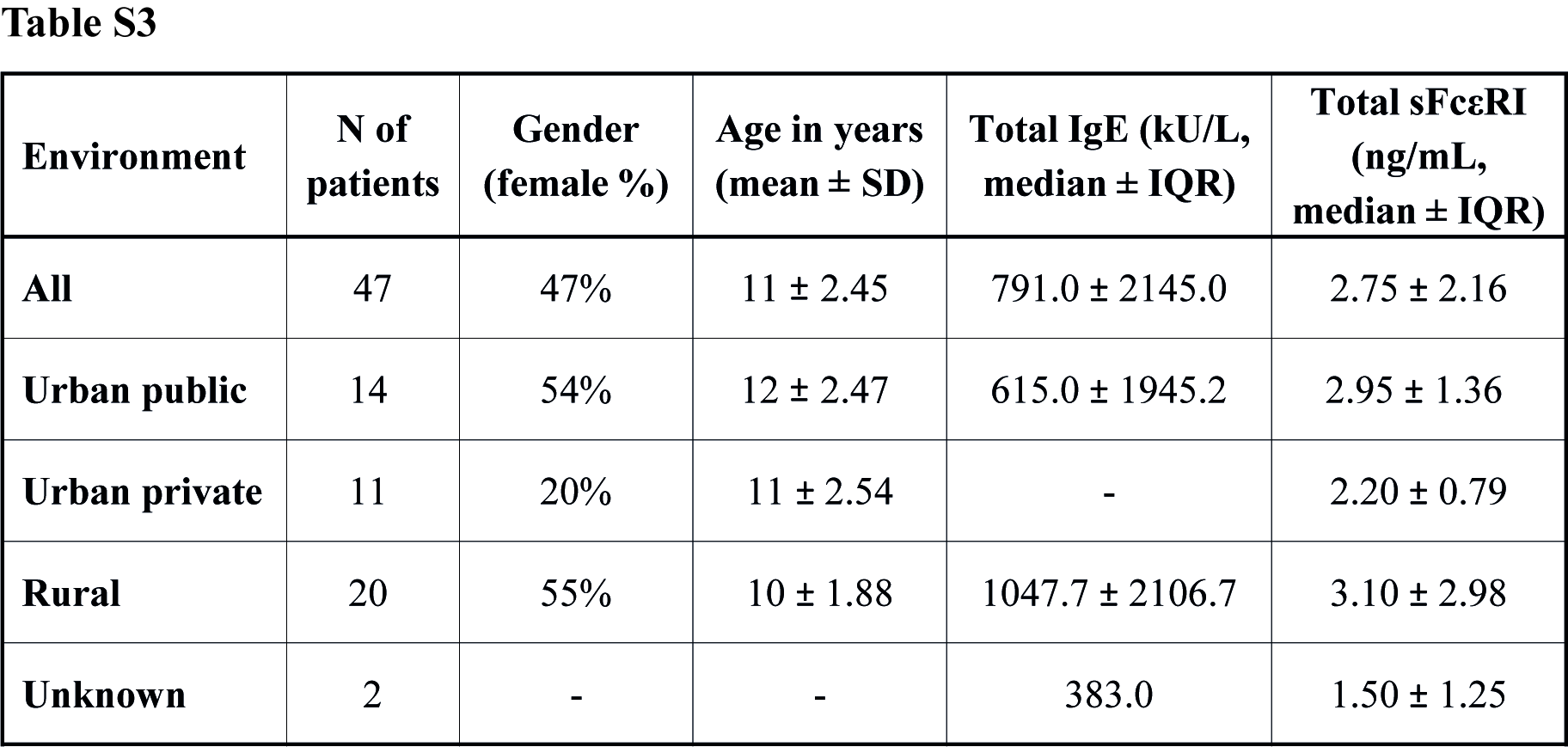
**

**Table S3: Patient characteristics of adult peanut-sensitized group from Ghana, West Africa.** SD: standard deviation; IQR: interquartile range.

**
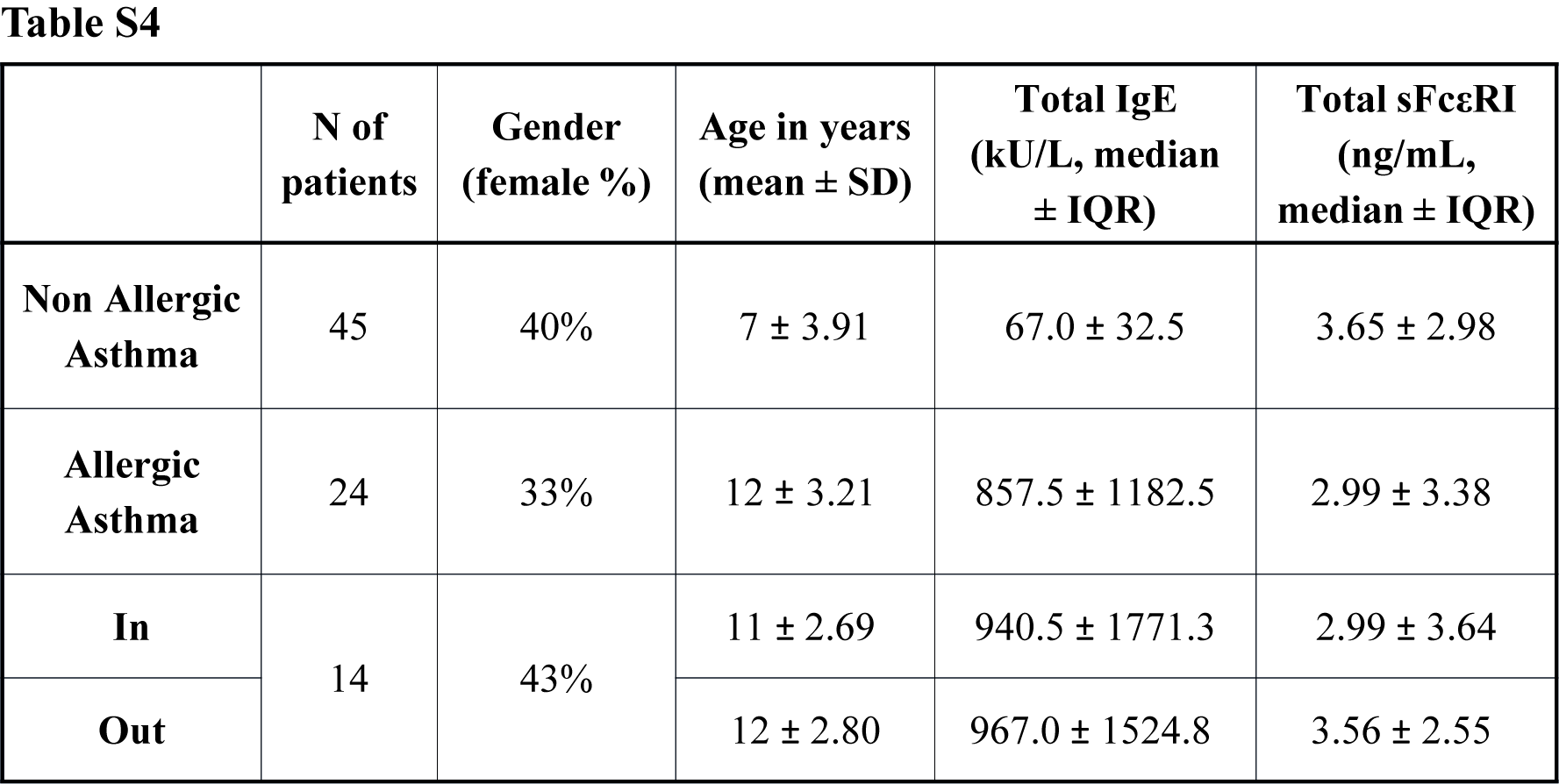
**

**Table S4: Patient characteristics of allergic asthmatic patients in and out of allergen season**. SD: standard deviation; IQR: interquartile range.
