# Supplementary figures and images for: Soluble FcɛRI: A biomarker for IgE‐mediated diseases
Source: Allergy. 2019 Mar 11;74(7):1381–4. doi: 10.1111/all.13734 (PMC6766993; doi:10.1111/all.13734)

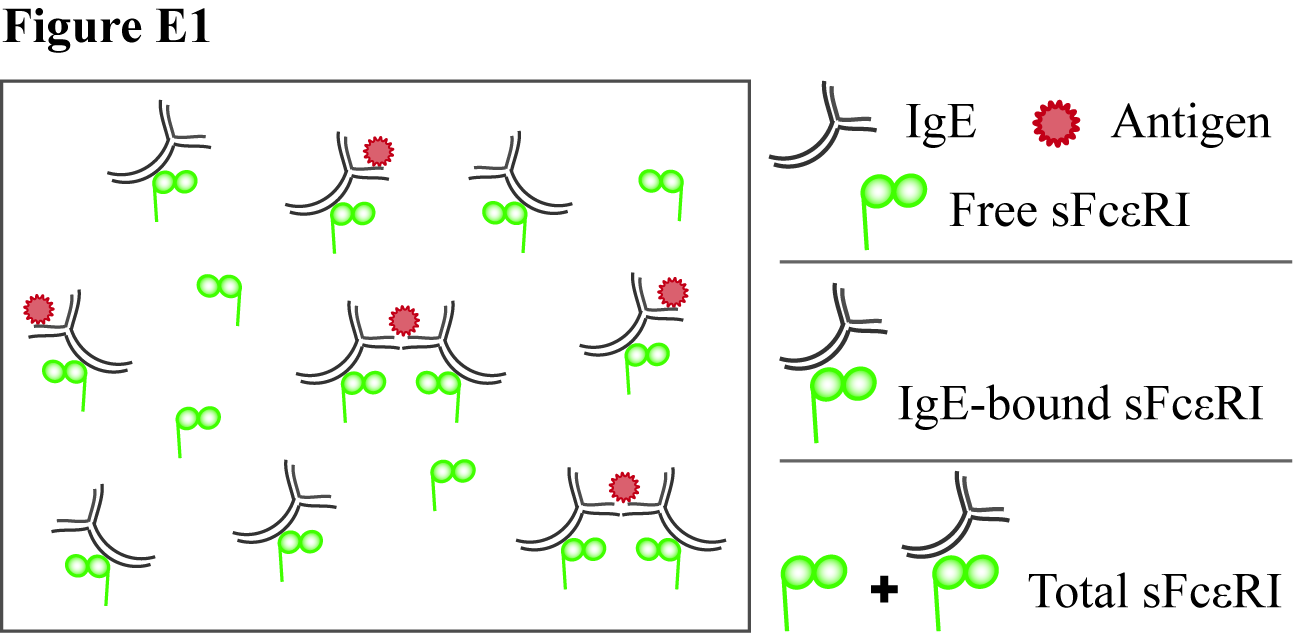

Supplement: Supplementary file 2 [file ALL-74-1381-s001.zip › FigureE1.tif]

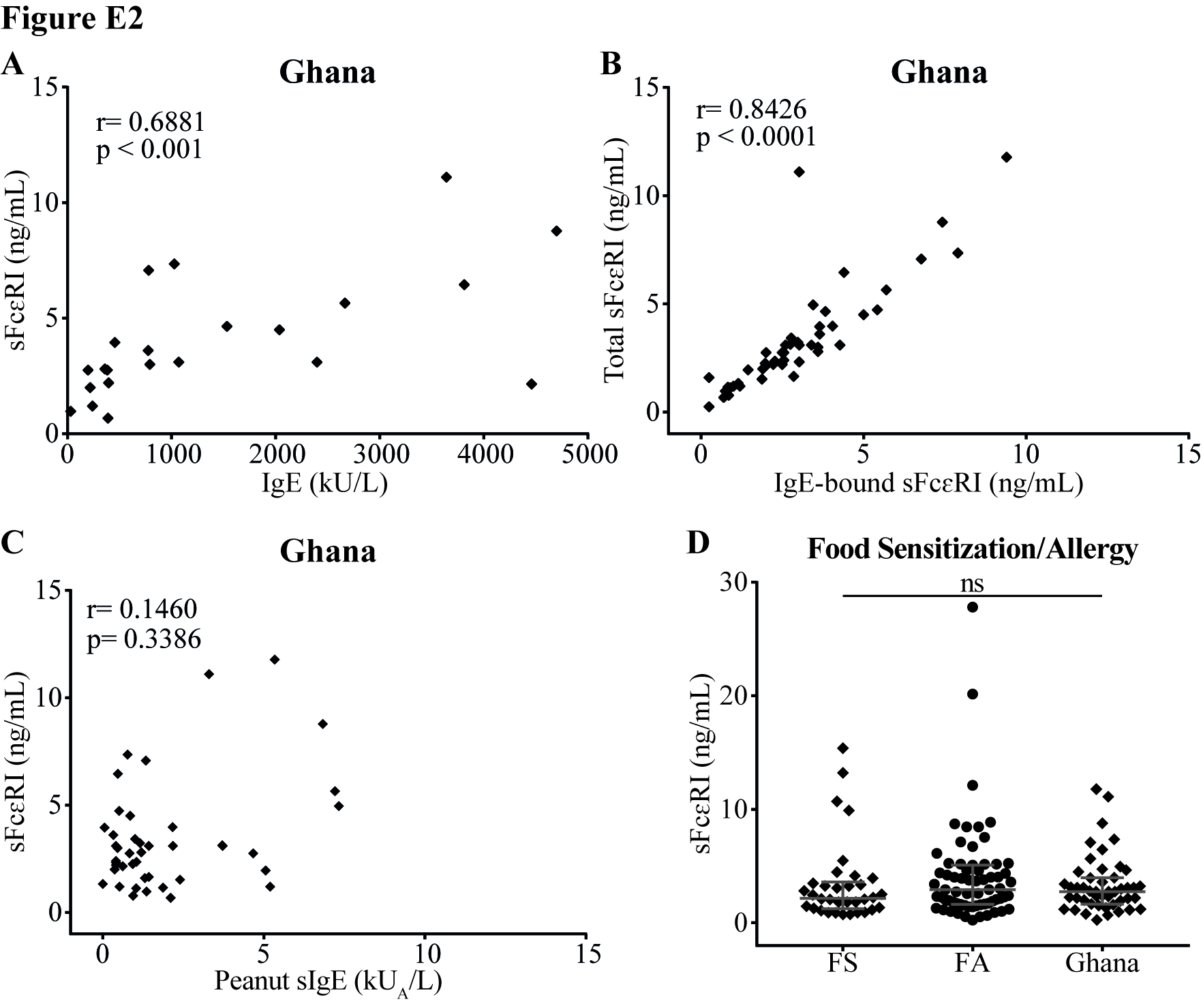

Supplement: Supplementary file 2 [file ALL-74-1381-s001.zip › FigureE2.tif]

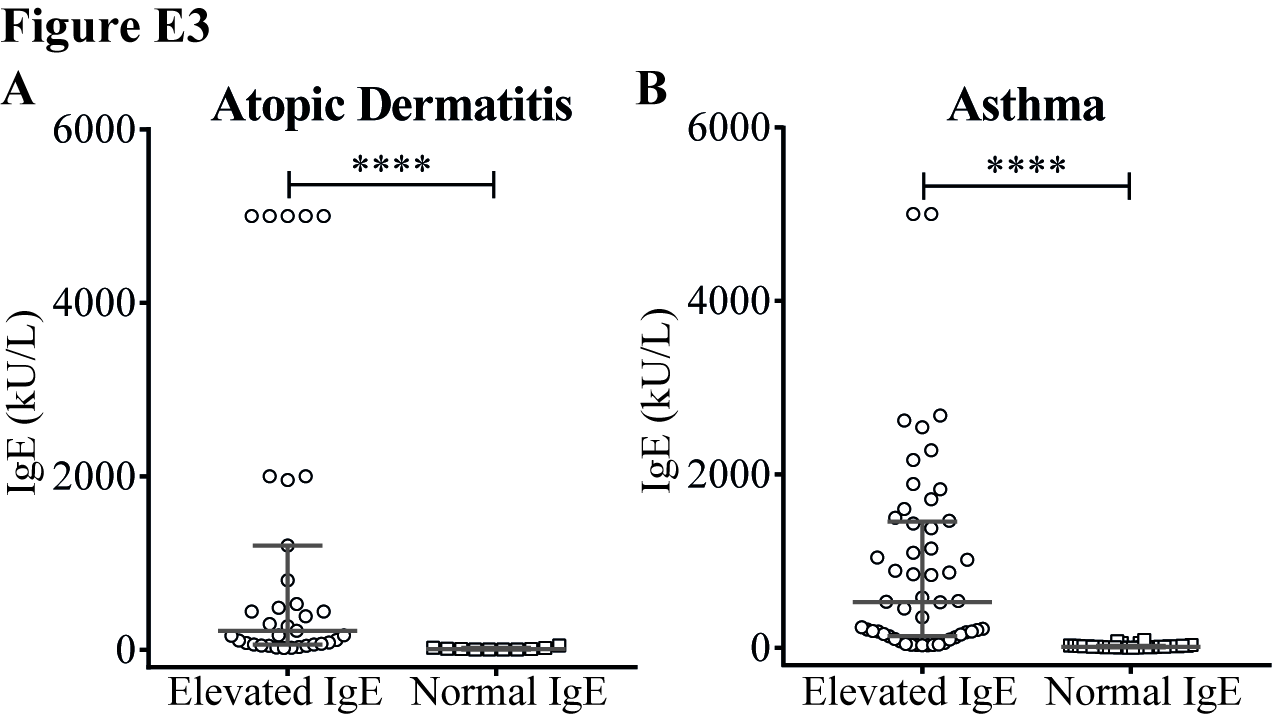

Supplement: Supplementary file 2 [file ALL-74-1381-s001.zip › FigureE3.tif]

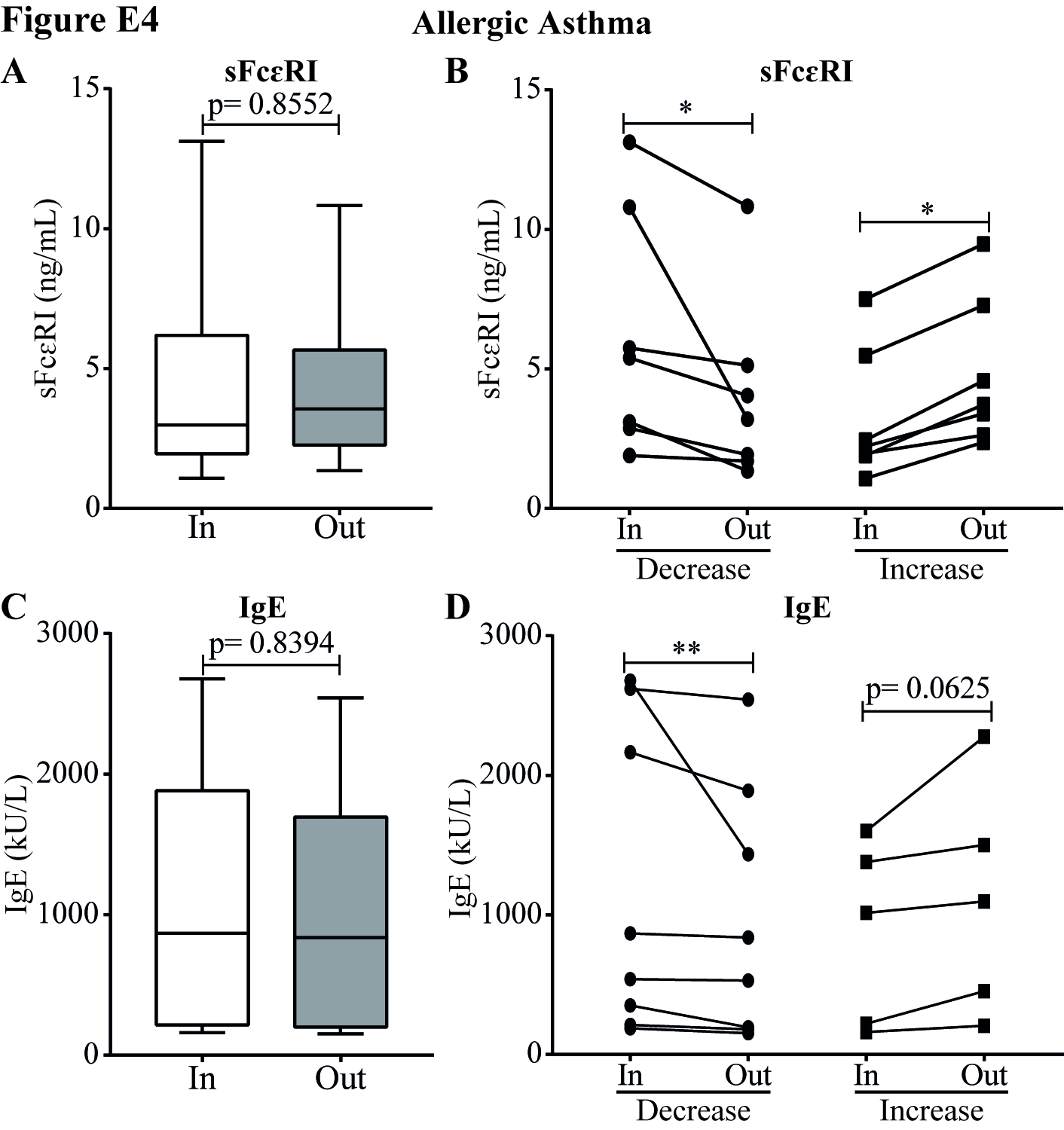

Supplement: Supplementary file 2 [file ALL-74-1381-s001.zip › FigureE4_Unmarked.tif]

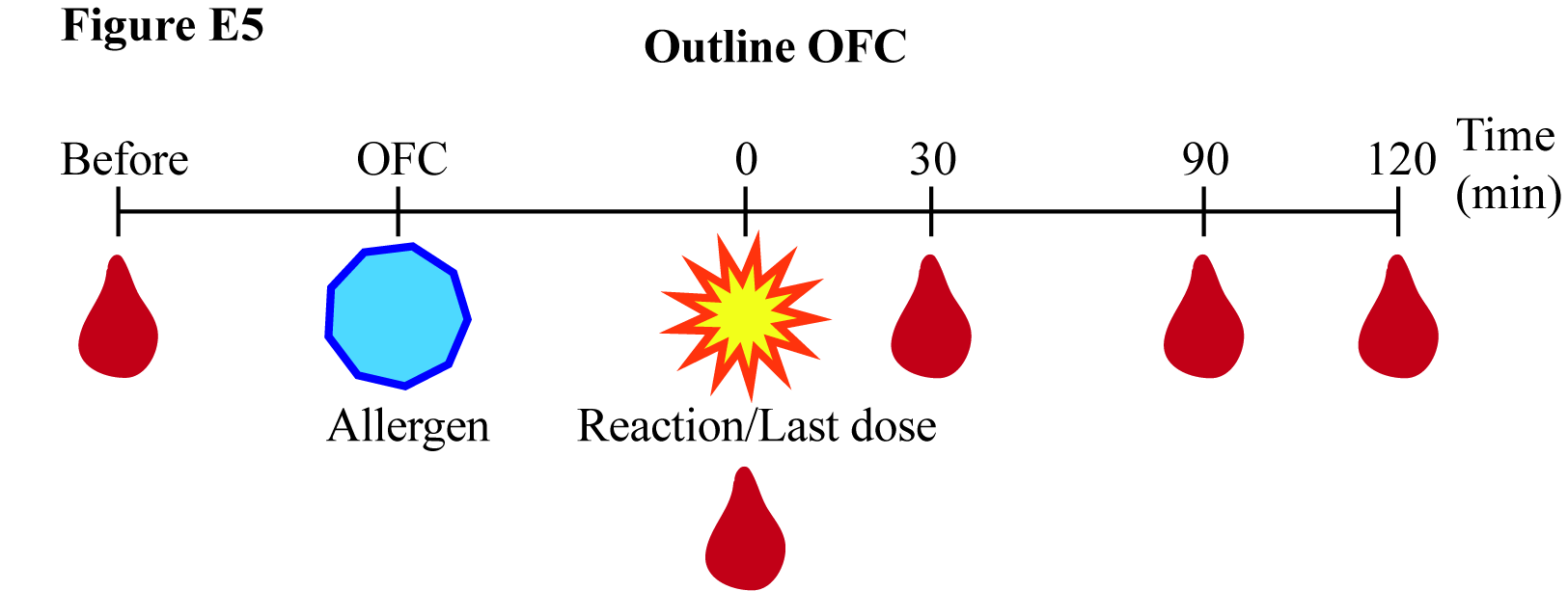

Supplement: Supplementary file 2 [file ALL-74-1381-s001.zip › FigureE5.tif]

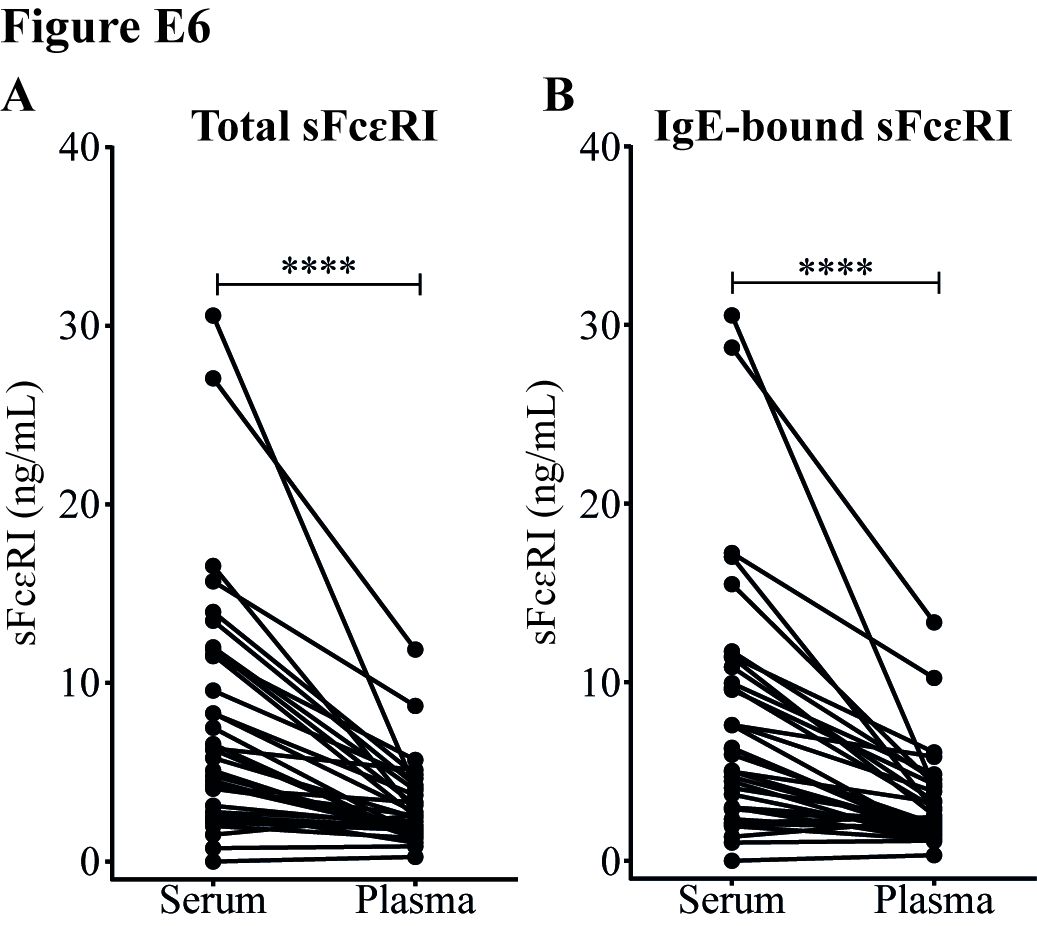

Supplement: Supplementary file 2 [file ALL-74-1381-s001.zip › FigureE6.tif]

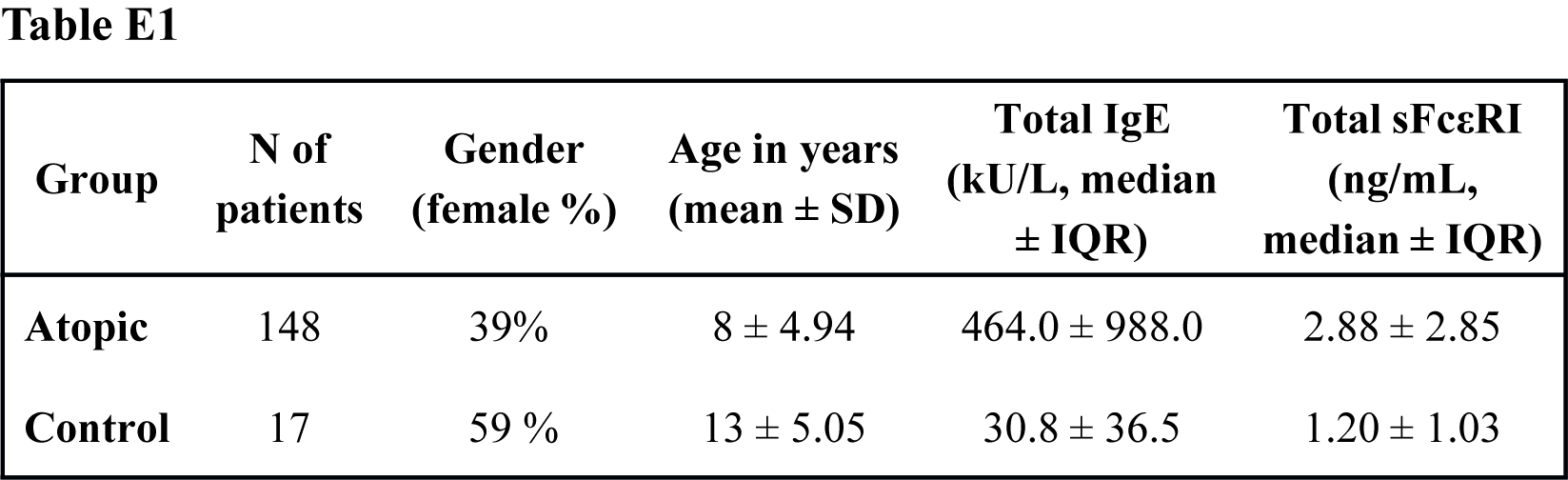

Supplement: Supplementary file 2 [file ALL-74-1381-s001.zip › TableE1.tif]

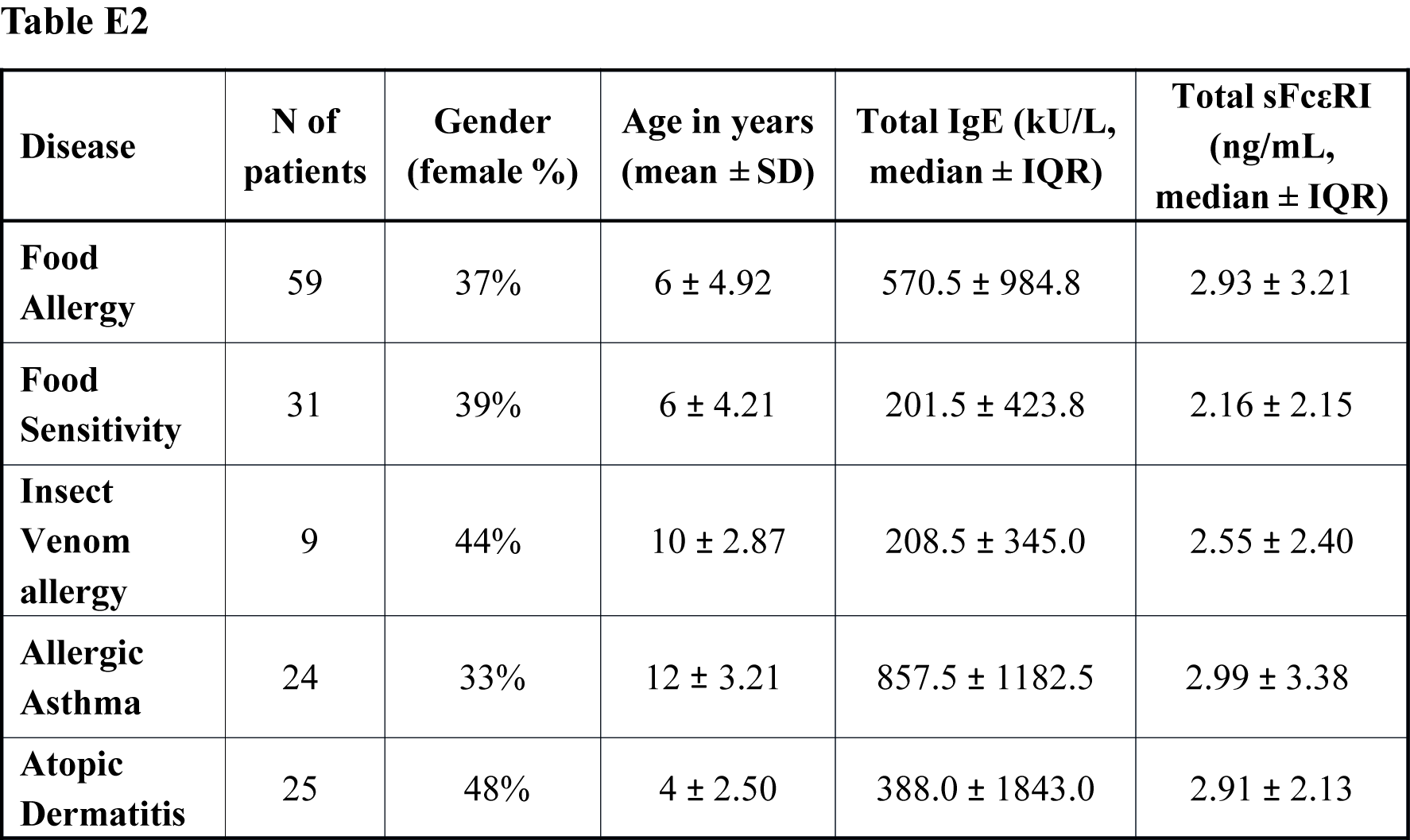

Supplement: Supplementary file 2 [file ALL-74-1381-s001.zip › TableE2.tif]

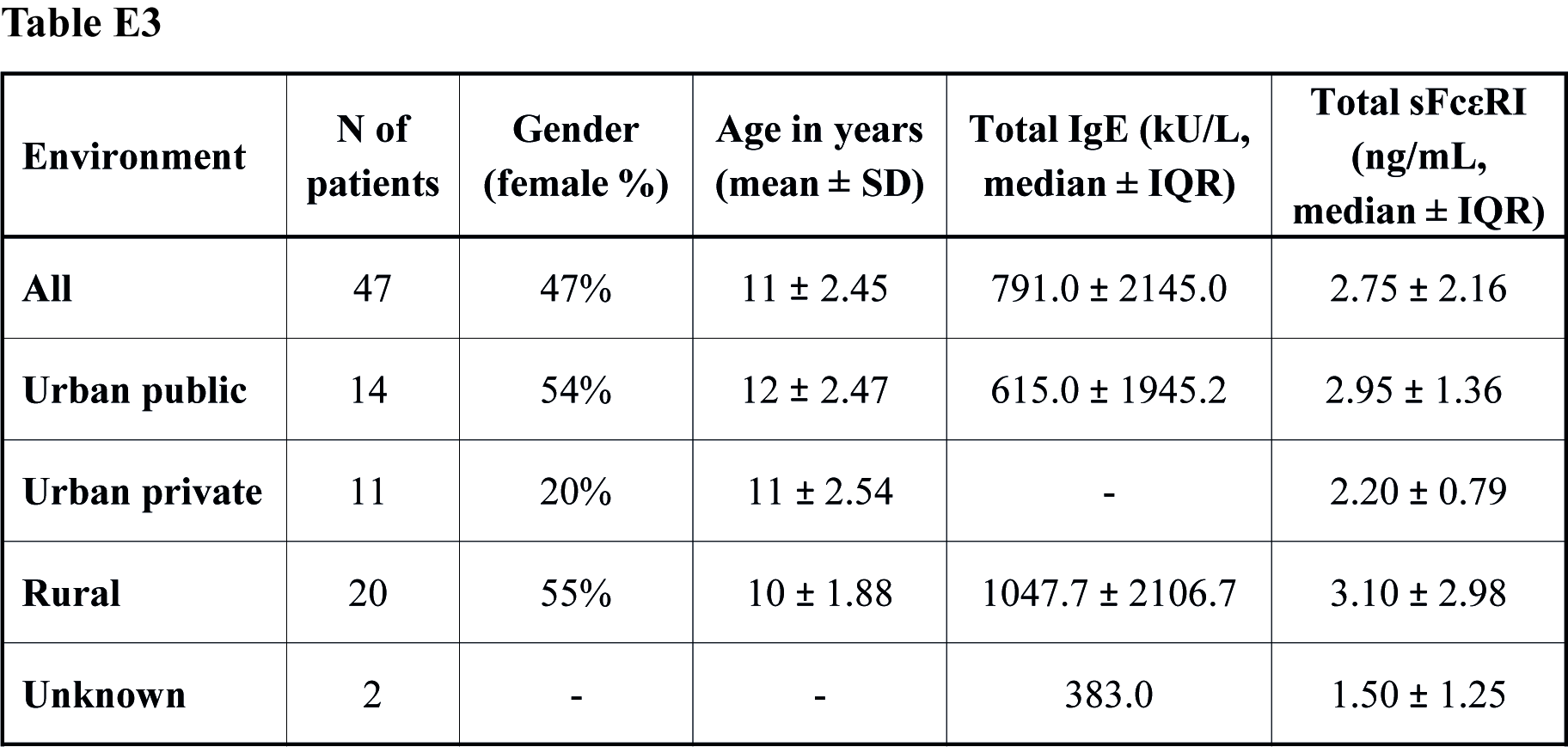

Supplement: Supplementary file 2 [file ALL-74-1381-s001.zip › TableE3.tif]

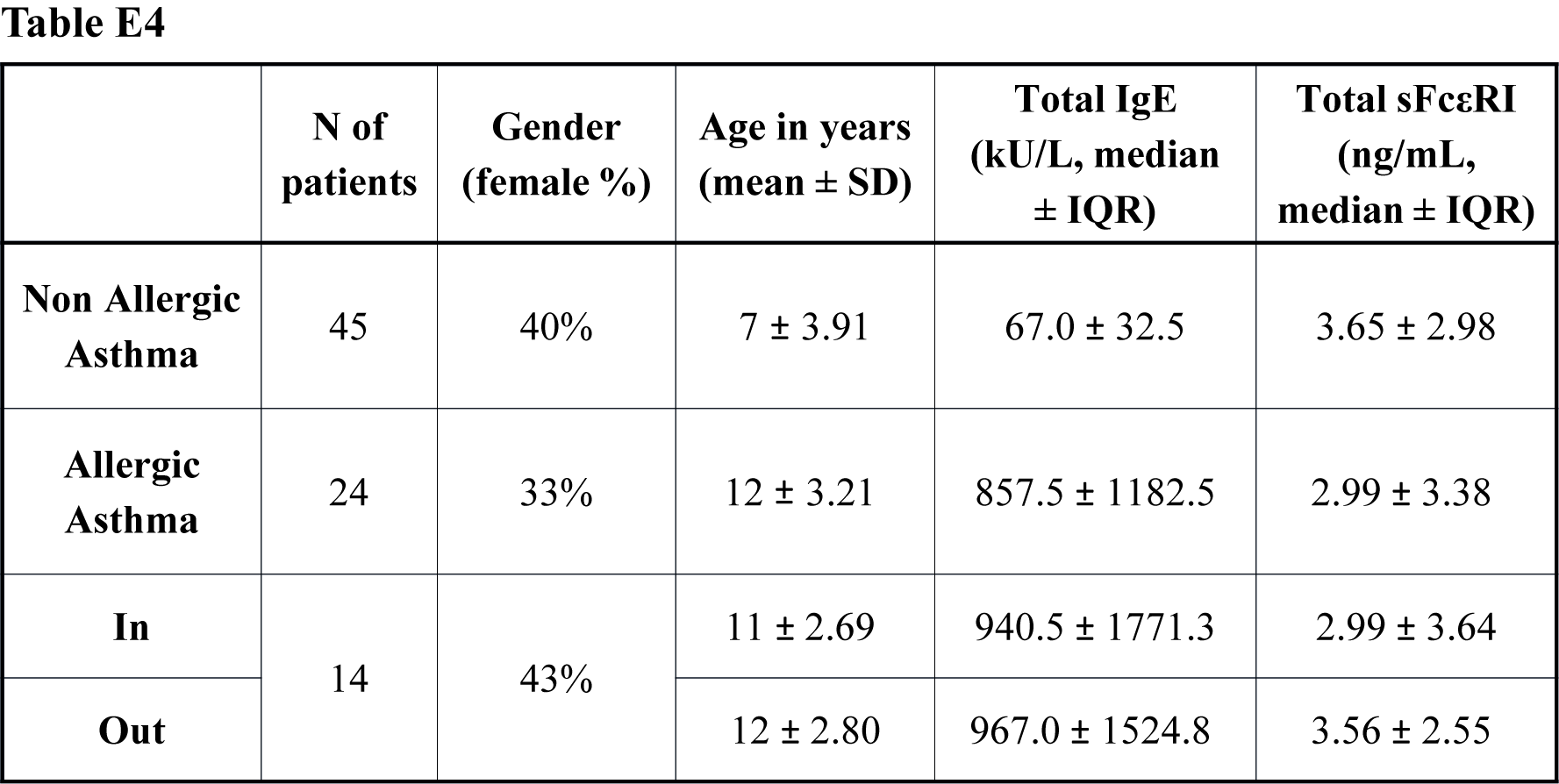

Supplement: Supplementary file 2 [file ALL-74-1381-s001.zip › TableE4.tif]
